# 4-Hy­droxy-*N*-methyl­benzamide

**DOI:** 10.1107/S1600536813009781

**Published:** 2013-04-17

**Authors:** Jerry P. Jasinski, Joel P. St. John, Ray J. Butcher, B. Narayana, H. S. Yathirajan, B. K. Sarojini

**Affiliations:** a Department of Chemistry, Keene State College, 229 Main Street, Keene, NH 03435-2001, USA; b Department of Chemistry, Howard University, 525 College Street NW, Washington, DC 20059, USA; c Department of Studies in Chemistry, Mangalore University, Mangalagangotri 574 199, India; d Department of Studies in Chemistry, University of Mysore, Manasagangotri, Mysore 570 006, India; e Department of Chemistry, P.A. College of Engineering, Nadupadavu, Mangalore 574 153, India

## Abstract

Three independent mol­ecules comprise the asymmetric unit of the title compound, C_8_H_9_NO_2_, in which the dihedral angles between the amide group and the benzene ring are 3.0 (2), 4.0 (3) and 3.3 (9)°. In the crystal, O—H⋯O hydrogen bonds and weak C—H⋯N inter­actions are observed, forming infinite chains along [101].

## Related literature

For background to the biological activity of aromatic amides, see: Saeed *et al.* (2008 ▶); Brunsveld *et al.* (2001 ▶); Prins *et al.* (2001 ▶). For the anti-emetic activity of *N*-substituted benzamides, see: Vega-Noverola *et al.* (1989 ▶). For related structures, see: Escalada *et al.* (2004 ▶); Pertlik (1992 ▶). For standard bond lengths, see: Allen *et al.* (1987 ▶).
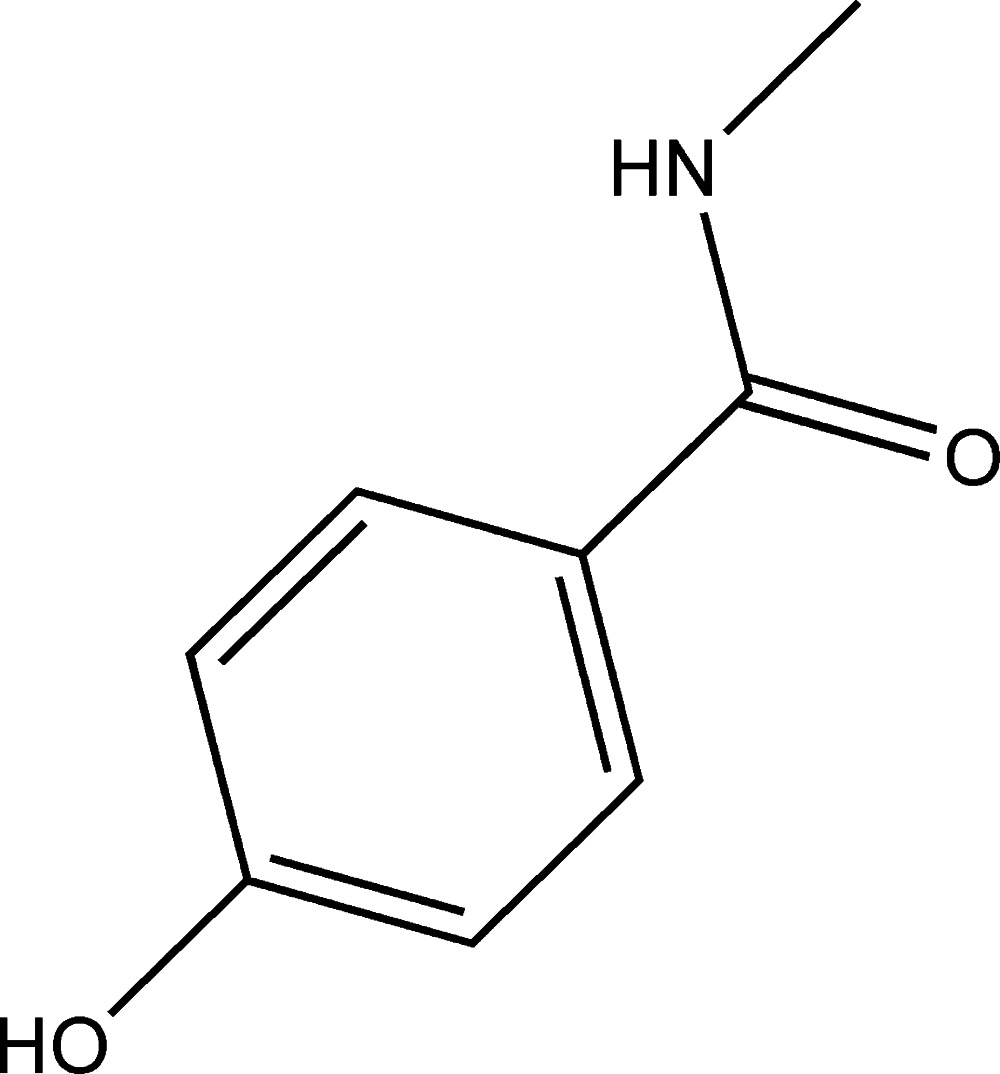


## Experimental

### 

#### Crystal data

C_8_H_9_NO_2_
*M* *_r_* = 151.16
Monoclinic, 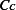

*a* = 13.576 (3) Å
*b* = 16.964 (3) Å
*c* = 11.025 (2) Å
β = 120.11 (3)°
*V* = 2196.5 (10) Å^3^
*Z* = 12
Mo *K*α radiation
μ = 0.10 mm^−1^
*T* = 100 K
0.42 × 0.28 × 0.22 mm

#### Data collection

Agilent Xcalibur diffractometer with a Ruby (Gemini Cu) detector
Absorption correction: multi-scan (*CrysAlis PRO* and *CrysAlis RED*; Agilent, 2012 ▶) *T*_min_ = 0.634, *T*_max_ = 1.000
4810 measured reflections
2802 independent reflections
2545 reflections with *I* > 2σ(*I*)
*R*_int_ = 0.015

#### Refinement

*R*[*F* ^2^ > 2σ(*F* ^2^)] = 0.059
*wR*(*F* ^2^) = 0.192
*S* = 1.10
2802 reflections
305 parameters
2 restraints
H-atom parameters constrained
Δρ_max_ = 0.58 e Å^−3^
Δρ_min_ = −0.56 e Å^−3^

### 

Data collection: *CrysAlis PRO* (Agilent, 2012 ▶); cell refinement: *CrysAlis PRO*; data reduction: *CrysAlis PRO*; program(s) used to solve structure: *SHELXS97* (Sheldrick, 2008 ▶); program(s) used to refine structure: *SHELXL2012* (Sheldrick, 2008 ▶); molecular graphics: *OLEX2* (Dolomanov *et al.*, 2009 ▶); software used to prepare material for publication: *OLEX2*.

## Supplementary Material

Crystal structure: contains datablock(s) global, I. DOI: 10.1107/S1600536813009781/hg5306sup1.cif

Structure factors: contains datablock(s) I. DOI: 10.1107/S1600536813009781/hg5306Isup2.hkl

Supplementary material file. DOI: 10.1107/S1600536813009781/hg5306Isup3.cml

Additional supplementary materials:  crystallographic information; 3D view; checkCIF report

## Figures and Tables

**Table 1:** Hydrogen-bond geometry (Å, °)

*D*—H⋯*A*	*D*—H	H⋯*A*	*D*⋯*A*	*D*—H⋯*A*
O1*A*—H1*A*⋯O2*C* ^i^	0.82	1.94	2.749 (5)	170
C2*A*—H2*A*⋯N1*A* ^ii^	0.93	2.66	3.267 (5)	124
C4*A*—H4*A*⋯N1*B* ^i^	0.93	2.60	3.371 (5)	141
O1*B*—H1*B*⋯O2*B* ^iii^	0.82	1.98	2.784 (5)	166
C2*B*—H2*B*⋯N1*C* ^iii^	0.93	2.63	3.404 (5)	142
O1*C*—H1*C*⋯O2*A*	0.82	1.96	2.750 (5)	163

Symmetry codes: (i) 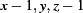; (ii) 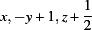; (iii) 
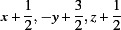
.

**Fig. 1.**
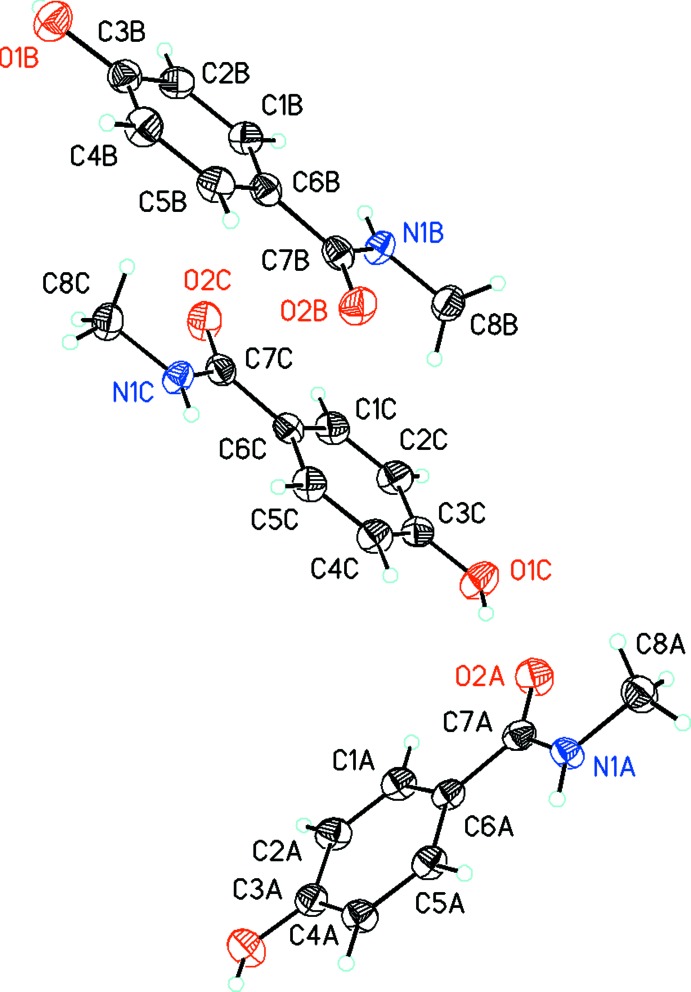
Molecular structure of the title compound showing the atom labeling scheme and 30% probability displacement ellipsoids of three independent molecules in the unit cell.

**Fig. 2.**
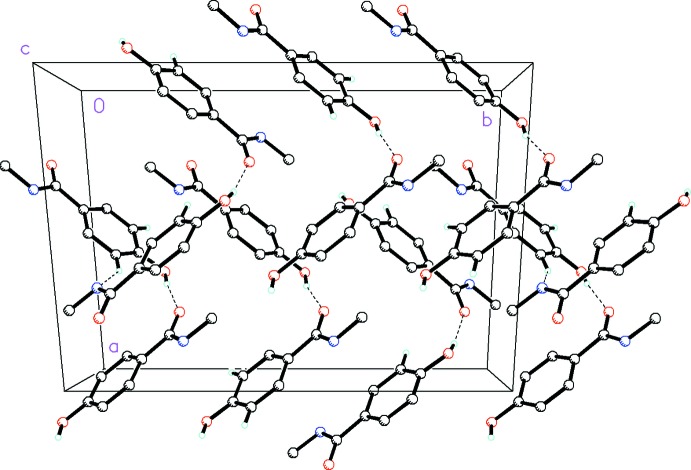
Packing diagram of the title compound viewed along the c axis. Dashed lines indicate O—H···O hydrogen bonds and weak C—H···O intermolecular interactions forming 1-D chains along (101). H atoms not involved in the hydrogen bonding and weak intermolecular interactions have been deleted for clarity.

**Fig. 3.**
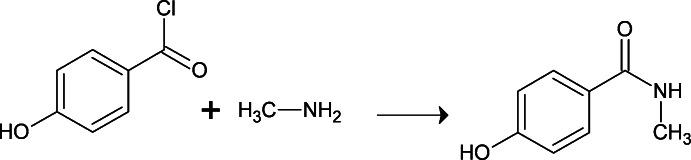
Reaction scheme for the synthesis of the title compound

## supplementary crystallographic information

### Comment

Aromatic amides have found extensive application in synthetic organic chemistry and have a wide range of biological activities (Saeed *et al.*, 2008, Brunsveld *et al.*, 2001; Prins *et al.*, 2001). Various N-substituted benzamides exhibit potent antiemetic activity (Vega-Noverola *et al.*, 1989). The crystal structure of N-methylbenzamide, viz., 2,3-dihydroxy-N-methylbenzamide monohydrate has been reported (Escalada *et al.*, 2004). Also the crystal structures of 2-hydroxy-N-methylbenzamide and 2-hydroxy-N-methylthiobenzamide have been published (Pertlik, 1992). In view of the importance of aromatic amides, we report the crystal structure of the title compound, C_8_H_9_NO_2_, (I).

In (I), three independent molecules (A, B. C) crystallize in the asymmetric unit (Fig. 1). Bond lengths are in normal ranges (Allen *et al.*, 1987). The dihedral angle between the amide group and the benzene ring is 3.0 (2)°, 4.0 (3)° and 3.3 (9)°, respectively. In the crystal, O—H···O hydrogen bonds and weak C—H···N intermolecular interactions are observed (Table 1) forming infinite 1-D chains along (101) and contribute to packing stability (Fig. 2). The closest intercentroid distance between two π-ring systems is 5.214 (6) Å.

### Experimental

4-Hydroxybenzoyl chloride (1.56 g, 0.01 mole) and methylamine (0.31 g, 0.01 mole) were dissolved in 20 ml methanol and stirred at room temperature for 3 h (Fig. 3). Then the reaction mass was poured into 50 ml ice cold water. The solid obtained was filtered and dried. Single crystals were grown from acetone by the slow evaporation method with a yield of 76%. (m.p. 395 K). Analytical data: Found (Calculated): C % : 63.54 (63.56); H% : 5.98 (6.00) ; N% : 9.21 (9.27).

### Refinement

All of the H atoms were placed in their calculated positions and then refined using the riding model with Atom—H lengths of 0.93Å (CH), 0.96Å (CH_3_), 0.86Å (NH) or 0.82Å (OH). Isotropic displacement parameters for these atoms were set to 1.2 (CH, NH) or 1.5 (CH_3_, OH) times *U*_eq_ of the parent atom. Aromatic/amide H refined with riding coordinates: N1A(H1AA), C1A(H1AB), C2A(H2A), C4A(H4A), C5A(H5A), N1B(H1BA), C1B(H1BB),C2B(H2B), C4B(H4B), C5B(H5B), N1C(H1CA), C1C(H1CB), C2C(H2C), C4C(H4C), C5C(H5C). Idealised Me refined as rotating group: C8A(H8AA,H8AB,H8AC), C8B(H8BA,H8BB,H8BC), C8C(H8CA,H8CB,H8CC). Idealised tetrahedral OH refined as rotating group: O1A(H1A), O1B(H1B), O1C(H1C).

### Crystal data

**Table:**

C_8_H_9_NO_2_	*F*(000) = 960
*M**_r_* = 151.16	*D*_x_ = 1.371 Mg m^−^^3^
Monoclinic, *C**c*	Mo *K*α radiation, λ = 0.71073 Å
*a* = 13.576 (3) Å	Cell parameters from 3483 reflections
*b* = 16.964 (3) Å	θ = 4.6–77.5°
*c* = 11.025 (2) Å	µ = 0.10 mm^−^^1^
β = 120.11 (3)°	*T* = 100 K
*V* = 2196.5 (10) Å^3^	Block, colourless
*Z* = 12	0.42 × 0.28 × 0.22 mm

### Data collection

**Table:**

Agilent Xcalibur diffractometer with a Ruby (Gemini Cu) detector	2545 reflections with *I* > 2σ(*I*)
Detector resolution: 10.5081 pixels mm^-1^	*R*_int_ = 0.015
ω scans	θ_max_ = 26.8°, θ_min_ = 2.1°
Absorption correction: multi-scan (*CrysAlis PRO* and *CrysAlis RED*; Agilent, 2012)	*h* = −17→12
*T*_min_ = 0.634, *T*_max_ = 1.000	*k* = −21→20
4810 measured reflections	*l* = −13→13
2802 independent reflections	

### Refinement

**Table:**

Refinement on *F*^2^	Hydrogen site location: inferred from neighbouring sites
Least-squares matrix: full	H-atom parameters constrained
*R*[*F*^2^ > 2σ(*F*^2^)] = 0.059	*w* = 1/[σ^2^(*F*_o_^2^) + (0.1461*P*)^2^ + 0.3673*P*], where *P* = (*F*_o_^2^ + 2*F*_c_^2^)/3
*wR*(*F*^2^) = 0.192	(Δ/σ)_max_ < 0.001
*S* = 1.10	Δρ_max_ = 0.58 e Å^−^^3^
2802 reflections	Δρ_min_ = −0.56 e Å^−^^3^
305 parameters	Extinction correction: *SHELXL*, Fc^*^=kFc[1+0.001xFc^2^λ^3^/sin(2θ)]^-1/4^
2 restraints	Extinction coefficient: 0.020 (6)

### Special details

**Table:**

Geometry. All esds (except the esd in the dihedral angle between two l.s. planes) are estimated using the full covariance matrix. The cell esds are taken into account individually in the estimation of esds in distances, angles and torsion angles; correlations between esds in cell parameters are only used when they are defined by crystal symmetry. An approximate (isotropic) treatment of cell esds is used for estimating esds involving l.s. planes.

### Fractional atomic coordinates and isotropic or equivalent isotropic displacement parameters (Å^2^)

**Table:**

	*x*	*y*	*z*	*U*_iso_*/*U*_eq_	
O1A	−0.1230 (3)	0.69501 (19)	0.1817 (4)	0.0616 (8)	
H1A	−0.1628	0.7033	0.0974	0.092*	
O2A	0.2475 (3)	0.42236 (17)	0.4097 (3)	0.0612 (8)	
N1A	0.1573 (2)	0.39940 (16)	0.1817 (3)	0.0404 (6)	
H1AA	0.1024	0.4109	0.0992	0.048*	
C1A	0.0968 (3)	0.5501 (2)	0.3666 (4)	0.0471 (8)	
H1AB	0.1499	0.5376	0.4589	0.056*	
C2A	0.0254 (4)	0.6130 (3)	0.3400 (4)	0.0504 (9)	
H2A	0.0306	0.6425	0.4141	0.060*	
C3A	−0.0548 (3)	0.6331 (2)	0.2026 (4)	0.0452 (8)	
C4A	−0.0623 (3)	0.5876 (2)	0.0918 (4)	0.0468 (8)	
H4A	−0.1158	0.6002	−0.0004	0.056*	
C5A	0.0102 (3)	0.5240 (2)	0.1201 (4)	0.0439 (8)	
H5A	0.0048	0.4938	0.0467	0.053*	
C6A	0.0917 (3)	0.5048 (2)	0.2590 (3)	0.0402 (7)	
C7A	0.1730 (3)	0.4393 (2)	0.2940 (4)	0.0431 (8)	
C8A	0.2365 (5)	0.3355 (3)	0.2037 (6)	0.0549 (9)	
H8AA	0.2520	0.3063	0.2861	0.082*	
H8AB	0.3062	0.3570	0.2156	0.082*	
H8AC	0.2034	0.3010	0.1239	0.082*	
O1B	1.1116 (3)	0.8638 (2)	0.7338 (3)	0.0617 (9)	
H1B	1.1397	0.8800	0.8145	0.093*	
O2B	0.7422 (3)	0.58944 (17)	0.5125 (3)	0.0576 (8)	
N1B	0.8321 (3)	0.56916 (17)	0.7436 (3)	0.0406 (7)	
H1BA	0.8845	0.5830	0.8261	0.049*	
C1B	0.9770 (3)	0.6941 (2)	0.7987 (4)	0.0455 (8)	
H1BB	0.9815	0.6651	0.8729	0.055*	
C2B	1.0491 (3)	0.7578 (2)	0.8255 (4)	0.0464 (9)	
H2B	1.1014	0.7717	0.9175	0.056*	
C3B	1.0433 (3)	0.8006 (2)	0.7158 (4)	0.0466 (9)	
C4B	0.9661 (4)	0.7791 (3)	0.5779 (5)	0.0540 (10)	
H4B	0.9628	0.8074	0.5038	0.065*	
C5B	0.8946 (3)	0.7156 (2)	0.5518 (4)	0.0486 (9)	
H5B	0.8434	0.7010	0.4598	0.058*	
C6B	0.8987 (3)	0.6735 (2)	0.6626 (4)	0.0422 (8)	
C7B	0.8164 (3)	0.6074 (2)	0.6300 (4)	0.0437 (9)	
C8B	0.7582 (4)	0.5039 (2)	0.7230 (5)	0.0562 (11)	
H8BA	0.7827	0.4587	0.6927	0.084*	
H8BB	0.6817	0.5173	0.6530	0.084*	
H8BC	0.7606	0.4919	0.8096	0.084*	
O1C	0.3742 (3)	0.4691 (2)	0.6850 (4)	0.0665 (9)	
H1C	0.3295	0.4643	0.6007	0.100*	
O2C	0.7454 (3)	0.74129 (17)	0.9069 (3)	0.0630 (8)	
N1C	0.6574 (3)	0.76016 (17)	0.6775 (3)	0.0433 (7)	
H1CA	0.6057	0.7459	0.5949	0.052*	
C1C	0.5934 (4)	0.6151 (3)	0.8683 (4)	0.0532 (9)	
H1CB	0.6448	0.6298	0.9601	0.064*	
C2C	0.5220 (4)	0.5522 (3)	0.8445 (4)	0.0558 (10)	
H2C	0.5255	0.5244	0.9193	0.067*	
C3C	0.4443 (3)	0.5305 (2)	0.7068 (4)	0.0484 (9)	
C4C	0.4387 (4)	0.5731 (2)	0.5946 (4)	0.0522 (9)	
H4C	0.3865	0.5590	0.5027	0.063*	
C5C	0.5114 (4)	0.6359 (2)	0.6212 (4)	0.0491 (9)	
H5C	0.5075	0.6644	0.5469	0.059*	
C6C	0.5906 (3)	0.6570 (2)	0.7589 (4)	0.0434 (8)	
C7C	0.6724 (3)	0.7228 (2)	0.7924 (4)	0.0463 (8)	
C8C	0.7327 (5)	0.8257 (2)	0.6993 (6)	0.0567 (10)	
H8CA	0.7403	0.8575	0.7756	0.085*	
H8CB	0.7016	0.8571	0.6156	0.085*	
H8CC	0.8061	0.8061	0.7211	0.085*	

### Atomic displacement parameters (Å^2^)

**Table:**

	*U*^11^	*U*^22^	*U*^33^	*U*^12^	*U*^13^	*U*^23^
O1A	0.068 (2)	0.0641 (17)	0.0477 (17)	0.0157 (15)	0.0253 (16)	−0.0002 (14)
O2A	0.0643 (19)	0.0652 (17)	0.0364 (15)	0.0055 (14)	0.0122 (13)	−0.0009 (13)
N1A	0.0413 (15)	0.0462 (13)	0.0254 (13)	0.0085 (12)	0.0107 (11)	0.0014 (11)
C1A	0.050 (2)	0.057 (2)	0.0305 (16)	−0.0027 (16)	0.0168 (16)	0.0005 (14)
C2A	0.051 (2)	0.062 (2)	0.0323 (19)	−0.0003 (18)	0.0161 (17)	−0.0064 (17)
C3A	0.047 (2)	0.0462 (18)	0.039 (2)	−0.0024 (14)	0.0196 (17)	0.0009 (14)
C4A	0.048 (2)	0.055 (2)	0.0309 (17)	0.0003 (17)	0.0146 (15)	0.0058 (15)
C5A	0.050 (2)	0.0504 (18)	0.0297 (18)	−0.0048 (15)	0.0187 (16)	−0.0044 (14)
C6A	0.0413 (17)	0.0466 (16)	0.0304 (17)	−0.0068 (14)	0.0161 (14)	−0.0005 (13)
C7A	0.0399 (17)	0.0477 (18)	0.0364 (18)	−0.0064 (13)	0.0152 (15)	0.0020 (14)
C8A	0.055 (2)	0.0538 (18)	0.050 (2)	0.0078 (16)	0.0221 (17)	0.0012 (16)
O1B	0.061 (2)	0.0612 (18)	0.053 (2)	−0.0118 (15)	0.0207 (17)	0.0036 (14)
O2B	0.0545 (17)	0.0564 (16)	0.0410 (17)	−0.0079 (13)	0.0084 (13)	−0.0068 (13)
N1B	0.0472 (17)	0.0416 (14)	0.0294 (14)	−0.0108 (13)	0.0165 (12)	−0.0029 (12)
C1B	0.046 (2)	0.0509 (19)	0.038 (2)	0.0015 (16)	0.0199 (17)	0.0043 (15)
C2B	0.0418 (19)	0.055 (2)	0.037 (2)	−0.0008 (16)	0.0159 (17)	−0.0020 (16)
C3B	0.044 (2)	0.0481 (19)	0.046 (2)	0.0016 (16)	0.0217 (18)	0.0039 (16)
C4B	0.060 (3)	0.058 (2)	0.039 (2)	0.0001 (19)	0.021 (2)	0.0109 (17)
C5B	0.048 (2)	0.055 (2)	0.0306 (18)	0.0001 (17)	0.0108 (17)	0.0027 (15)
C6B	0.0440 (19)	0.0419 (16)	0.038 (2)	0.0047 (14)	0.0188 (17)	0.0015 (14)
C7B	0.048 (2)	0.0435 (18)	0.036 (2)	0.0085 (15)	0.0189 (17)	0.0025 (14)
C8B	0.057 (2)	0.049 (2)	0.065 (3)	−0.0082 (19)	0.032 (2)	−0.009 (2)
O1C	0.069 (2)	0.0711 (19)	0.0519 (19)	−0.0204 (17)	0.0249 (16)	0.0013 (15)
O2C	0.070 (2)	0.0558 (15)	0.0412 (17)	−0.0042 (14)	0.0114 (14)	0.0022 (13)
N1C	0.0464 (16)	0.0442 (13)	0.0334 (14)	−0.0093 (12)	0.0156 (12)	−0.0013 (12)
C1C	0.053 (2)	0.059 (2)	0.0360 (19)	0.0029 (17)	0.0141 (17)	0.0029 (16)
C2C	0.055 (3)	0.066 (2)	0.037 (2)	−0.0010 (19)	0.0153 (19)	0.0130 (18)
C3C	0.044 (2)	0.0509 (19)	0.045 (2)	0.0008 (15)	0.0184 (17)	0.0005 (16)
C4C	0.053 (2)	0.059 (2)	0.037 (2)	0.0008 (18)	0.0176 (18)	−0.0027 (16)
C5C	0.055 (2)	0.053 (2)	0.034 (2)	0.0017 (17)	0.0187 (18)	0.0030 (15)
C6C	0.0439 (18)	0.0445 (16)	0.039 (2)	0.0077 (15)	0.0185 (16)	0.0020 (14)
C7C	0.046 (2)	0.0417 (17)	0.047 (2)	0.0048 (14)	0.0198 (17)	−0.0018 (14)
C8C	0.063 (3)	0.0435 (18)	0.066 (3)	−0.0039 (17)	0.034 (2)	0.0017 (18)

### Geometric parameters (Å, º)

**Table:**

O1A—H1A	0.8200	C2B—C3B	1.379 (5)
O1A—C3A	1.341 (5)	C3B—C4B	1.395 (6)
O2A—C7A	1.199 (5)	C4B—H4B	0.9300
N1A—H1AA	0.8600	C4B—C5B	1.379 (6)
N1A—C7A	1.331 (5)	C5B—H5B	0.9300
N1A—C8A	1.460 (5)	C5B—C6B	1.393 (5)
C1A—H1AB	0.9300	C6B—C7B	1.493 (5)
C1A—C2A	1.371 (6)	C8B—H8BA	0.9600
C1A—C6A	1.386 (5)	C8B—H8BB	0.9600
C2A—H2A	0.9300	C8B—H8BC	0.9600
C2A—C3A	1.394 (6)	O1C—H1C	0.8200
C3A—C4A	1.405 (5)	O1C—C3C	1.349 (5)
C4A—H4A	0.9300	O2C—C7C	1.191 (5)
C4A—C5A	1.386 (5)	N1C—H1CA	0.8600
C5A—H5A	0.9300	N1C—C7C	1.338 (5)
C5A—C6A	1.405 (5)	N1C—C8C	1.446 (5)
C6A—C7A	1.474 (5)	C1C—H1CB	0.9300
C8A—H8AA	0.9600	C1C—C2C	1.376 (6)
C8A—H8AB	0.9600	C1C—C6C	1.385 (5)
C8A—H8AC	0.9600	C2C—H2C	0.9300
O1B—H1B	0.8200	C2C—C3C	1.395 (6)
O1B—C3B	1.364 (5)	C3C—C4C	1.402 (6)
O2B—C7B	1.215 (5)	C4C—H4C	0.9300
N1B—H1BA	0.8600	C4C—C5C	1.380 (6)
N1B—C7B	1.330 (5)	C5C—H5C	0.9300
N1B—C8B	1.434 (5)	C5C—C6C	1.397 (5)
C1B—H1BB	0.9300	C6C—C7C	1.484 (5)
C1B—C2B	1.387 (6)	C8C—H8CA	0.9600
C1B—C6B	1.380 (6)	C8C—H8CB	0.9600
C2B—H2B	0.9300	C8C—H8CC	0.9600
			
C3A—O1A—H1A	109.5	C4B—C5B—H5B	119.9
C7A—N1A—H1AA	121.2	C4B—C5B—C6B	120.2 (4)
C7A—N1A—C8A	117.5 (4)	C6B—C5B—H5B	119.9
C8A—N1A—H1AA	121.2	C1B—C6B—C5B	119.6 (4)
C2A—C1A—H1AB	119.2	C1B—C6B—C7B	121.8 (3)
C2A—C1A—C6A	121.5 (4)	C5B—C6B—C7B	118.6 (4)
C6A—C1A—H1AB	119.2	O2B—C7B—N1B	122.5 (4)
C1A—C2A—H2A	119.8	O2B—C7B—C6B	124.4 (4)
C1A—C2A—C3A	120.4 (3)	N1B—C7B—C6B	113.1 (3)
C3A—C2A—H2A	119.8	N1B—C8B—H8BA	109.5
O1A—C3A—C2A	118.2 (3)	N1B—C8B—H8BB	109.5
O1A—C3A—C4A	122.6 (4)	N1B—C8B—H8BC	109.5
C2A—C3A—C4A	119.2 (3)	H8BA—C8B—H8BB	109.5
C3A—C4A—H4A	120.1	H8BA—C8B—H8BC	109.5
C5A—C4A—C3A	119.8 (4)	H8BB—C8B—H8BC	109.5
C5A—C4A—H4A	120.1	C3C—O1C—H1C	109.5
C4A—C5A—H5A	119.7	C7C—N1C—H1CA	121.7
C4A—C5A—C6A	120.6 (3)	C7C—N1C—C8C	116.6 (4)
C6A—C5A—H5A	119.7	C8C—N1C—H1CA	121.7
C1A—C6A—C5A	118.5 (3)	C2C—C1C—H1CB	119.2
C1A—C6A—C7A	119.0 (3)	C2C—C1C—C6C	121.6 (4)
C5A—C6A—C7A	122.5 (3)	C6C—C1C—H1CB	119.2
O2A—C7A—N1A	121.6 (4)	C1C—C2C—H2C	120.4
O2A—C7A—C6A	125.4 (4)	C1C—C2C—C3C	119.2 (4)
N1A—C7A—C6A	113.0 (3)	C3C—C2C—H2C	120.4
N1A—C8A—H8AA	109.5	O1C—C3C—C2C	118.6 (4)
N1A—C8A—H8AB	109.5	O1C—C3C—C4C	121.3 (4)
N1A—C8A—H8AC	109.5	C2C—C3C—C4C	120.1 (4)
H8AA—C8A—H8AB	109.5	C3C—C4C—H4C	120.2
H8AA—C8A—H8AC	109.5	C5C—C4C—C3C	119.6 (4)
H8AB—C8A—H8AC	109.5	C5C—C4C—H4C	120.2
C3B—O1B—H1B	109.5	C4C—C5C—H5C	119.7
C7B—N1B—H1BA	121.3	C4C—C5C—C6C	120.6 (3)
C7B—N1B—C8B	117.3 (3)	C6C—C5C—H5C	119.7
C8B—N1B—H1BA	121.3	C1C—C6C—C5C	119.0 (4)
C2B—C1B—H1BB	119.8	C1C—C6C—C7C	118.6 (4)
C6B—C1B—H1BB	119.8	C5C—C6C—C7C	122.4 (3)
C6B—C1B—C2B	120.4 (4)	O2C—C7C—N1C	122.0 (4)
C1B—C2B—H2B	120.0	O2C—C7C—C6C	125.6 (4)
C3B—C2B—C1B	120.0 (4)	N1C—C7C—C6C	112.4 (3)
C3B—C2B—H2B	120.0	N1C—C8C—H8CA	109.5
O1B—C3B—C2B	123.4 (4)	N1C—C8C—H8CB	109.5
O1B—C3B—C4B	116.7 (4)	N1C—C8C—H8CC	109.5
C2B—C3B—C4B	119.9 (4)	H8CA—C8C—H8CB	109.5
C3B—C4B—H4B	120.1	H8CA—C8C—H8CC	109.5
C5B—C4B—C3B	119.9 (4)	H8CB—C8C—H8CC	109.5
C5B—C4B—H4B	120.1		
			
O1A—C3A—C4A—C5A	−179.7 (3)	C3B—C4B—C5B—C6B	0.5 (6)
C1A—C2A—C3A—O1A	179.9 (4)	C4B—C5B—C6B—C1B	−1.8 (6)
C1A—C2A—C3A—C4A	0.6 (6)	C4B—C5B—C6B—C7B	177.3 (3)
C1A—C6A—C7A—O2A	−2.4 (5)	C5B—C6B—C7B—O2B	−3.0 (6)
C1A—C6A—C7A—N1A	178.5 (3)	C5B—C6B—C7B—N1B	176.9 (4)
C2A—C1A—C6A—C5A	−0.7 (5)	C6B—C1B—C2B—C3B	−0.5 (6)
C2A—C1A—C6A—C7A	178.3 (3)	C8B—N1B—C7B—O2B	0.8 (6)
C2A—C3A—C4A—C5A	−0.3 (5)	C8B—N1B—C7B—C6B	−179.1 (3)
C3A—C4A—C5A—C6A	−0.4 (5)	O1C—C3C—C4C—C5C	179.6 (4)
C4A—C5A—C6A—C1A	0.9 (5)	C1C—C2C—C3C—O1C	−179.6 (4)
C4A—C5A—C6A—C7A	−178.0 (3)	C1C—C2C—C3C—C4C	−0.6 (6)
C5A—C6A—C7A—O2A	176.5 (4)	C1C—C6C—C7C—O2C	3.7 (6)
C5A—C6A—C7A—N1A	−2.6 (4)	C1C—C6C—C7C—N1C	−177.5 (3)
C6A—C1A—C2A—C3A	0.0 (6)	C2C—C1C—C6C—C5C	1.6 (6)
C8A—N1A—C7A—O2A	−1.9 (6)	C2C—C1C—C6C—C7C	−178.3 (4)
C8A—N1A—C7A—C6A	177.2 (3)	C2C—C3C—C4C—C5C	0.6 (6)
O1B—C3B—C4B—C5B	179.8 (4)	C3C—C4C—C5C—C6C	0.4 (6)
C1B—C2B—C3B—O1B	−179.7 (4)	C4C—C5C—C6C—C1C	−1.5 (6)
C1B—C2B—C3B—C4B	−0.9 (6)	C4C—C5C—C6C—C7C	178.4 (3)
C1B—C6B—C7B—O2B	176.1 (4)	C5C—C6C—C7C—O2C	−176.2 (4)
C1B—C6B—C7B—N1B	−3.9 (5)	C5C—C6C—C7C—N1C	2.7 (5)
C2B—C1B—C6B—C5B	1.8 (6)	C6C—C1C—C2C—C3C	−0.5 (7)
C2B—C1B—C6B—C7B	−177.3 (3)	C8C—N1C—C7C—O2C	−1.5 (6)
C2B—C3B—C4B—C5B	0.9 (6)	C8C—N1C—C7C—C6C	179.6 (3)

### Hydrogen-bond geometry (Å, º)

**Table:**

*D*—H···*A*	*D*—H	H···*A*	*D*···*A*	*D*—H···*A*
O1*A*—H1*A*···O2*C*^i^	0.82	1.94	2.749 (5)	170
C2*A*—H2*A*···N1*A*^ii^	0.93	2.66	3.267 (5)	124
C4*A*—H4*A*···N1*B*^i^	0.93	2.60	3.371 (5)	141
O1*B*—H1*B*···O2*B*^iii^	0.82	1.98	2.784 (5)	166
C2*B*—H2*B*···N1*C*^iii^	0.93	2.63	3.404 (5)	142
O1*C*—H1*C*···O2*A*	0.82	1.96	2.750 (5)	163

Symmetry codes: (i) *x*−1, *y*, *z*−1; (ii) *x*, −*y*+1, *z*+1/2; (iii) *x*+1/2, −*y*+3/2, *z*+1/2.
